# Development of Phage-Based Single Chain Fv Antibody Reagents for Detection of *Yersinia pestis*

**DOI:** 10.1371/journal.pone.0027756

**Published:** 2011-12-08

**Authors:** Antonietta M. Lillo, Joanne E. Ayriss, Yulin Shou, Steven W. Graves, Andrew R. M. Bradbury

**Affiliations:** Bioscience Division, Los Alamos National Laboratory, Los Alamos, New Mexico, United States of America; Louisiana State University, United States of America

## Abstract

**Background:**

Most *Yersinia pestis* strains are known to express a capsule-like antigen, fraction 1 (F1)^.^ F1 is encoded by the caf1 gene located on the large 100-kb pFra plasmid, which is found in *Y. pestis* but not in closely related species such as *Yersinia enterocolytica* and *Yersinia pseudotuberculosis*. In order to find antibodies specifically binding to *Y. pestis* we screened a large single chain Fv antibody fragment (scFv) phage display library using purified F1 antigen as a selection target. Different forms of the selected antibodies were used to establish assays for recombinant F1 antigen and *Y. pestis* detection.

**Methods:**

Phage antibody panning was performed against F1 in an automated fashion using the Kingfisher magnetic bead system. Selected scFvs were screened for F1-binding specificity by one-step alkaline phosphatase enzyme linked immunosorbant assay (ELISA), and assayed for binding to recombinant antigen and/or *Y. pestis* by flow cytometry and whole-cell ELISA.

**Results:**

Seven of the eight selected scFvs were shown to specifically bind both recombinant F1 and a panel of F1-positive *Yersinia* cells. The majority of the soluble scFvs were found to be difficult to purify, unstable and prone to cross-reactivity with F1-negative *Yersinia* strains, whereas phage displayed scFvs were found to be easy to purify/label and remarkably stable. Furthermore direct fluorescent labeling of phage displaying scFv allowed for an easy one-step flow cytometry assay. Slight cross-reactivity was observed when fixed cells were used in ELISA.

**Conclusions:**

Our high throughput methods of selection and screening allowed for time and cost effective discovery of seven scFvs specifically binding *Y. pestis* F1 antigen. We describe implementation of different methods for phage-based immunoassay. Based on the success of these methods and the proven stability of phage, we indicate that the use of phage-displayed, rather than phage-free proteins, might generally overcome the shortcomings of scFv antibodies.

## Introduction

*Yersinia pestis* is a gram-negative, non-spore-forming bacterium belonging to the family Enterobacteriaceae that is known to have evolved from the enteric pathogen *Yersinia pseudotuberculosis* approximately 20,000 years ago [1]. Among the eleven true *Yersinia* species three are pathogenic to humans; *Yersinia pestis*, *Yersinia pseudotuberculosis* and *Yersinia enterocolytica*, while all others are harmless [2]. *Y. pestis* is the causative agent of the plague; an illness that manifests itself in bubonic, pneumonic or septicaemic forms that has resulted in the death of an estimated 200 million people throughout history [2]. Once aerosolized, the infectious agent can be dispersed and transmitted via inhalation causing pneumonic plague, the least common but most virulent form, which has the potential to cause high rates of morbidity and mortality in humans.

Currently, *Y. pestis* is listed as a National Institute of Allergy and Infectious Disease, Biodefense Category A Priority pathogen (http://www3.niaid.nih.gov/topics/BiodefenseRelated/Biodefense/research/CatA.htm), and is viewed as a high-priority agent that poses a risk to national security because it is relatively easy to acquire from the environment, and can be effectively dried and converted into an aerosol form. Therefore the development of methods for *Y. pestis* detection is relevant to public health and biosurveillance.

Specific detection of a microorganism is based on recognition of a genotypical or phenotypical feature unique to that microorganism for which nucleic acid-based or immunological detection technologies are adopted respectively. Immunological detection is inherently more rapid and therefore whenever possible preferable to nucleic acid-based detection since minimal sample preparation is required. Generally the development of immunoassays uses polyclonal or monoclonal full-length antibodies (mAb). The procedure to generate mAbs is time consuming (2–3 months), labor intensive and requires immunization. Furthermore, all procedures to derive mAbs with specific recognition characteristics (e.g. recognizes target A, but not closely related target B) are carried out at the screening stage. Because of the limitations of the number of clones which can be grown up this limits the number of mAbs that can be assessed and the specificities that can be obtained.

In recent years, antibody phage display has become a very popular method to isolate specific antibodies, bypassing hybridoma technology and even the need for immunization. In general, libraries are made up of either single chain Fv (scFv) or Fab fragments, and comprise billions of different clones, from which specific binders can be isolated by recursive selection cycles. One of the advantages of using phage antibody libraries is that several antibody fragments, binding to different epitopes, are usually selected, providing a greater likelihood that useful binders will be obtained. Traditional screening methods involve ELISA, a time and cost ineffective way to analyze a large number of clones for binding specificity. We have recently reported a multiplex flow cytometry screening method [3] which allows the analysis of hundreds of clones for binding specificity by simultaneously assaying interaction with the target antigen and a vast array of negative controls. This method has led to the discovery of sets of scFvs with exquisite binding specificity to the target antigen and potentially binding multiple epitopes within the antigen. Using such *in vitro* methods for antibody fragments discovery has been extremely successful, however the use of scFv as reagents in research, diagnosis or detection has been limited by handling issues: low production levels, aggregation and poor stability in long-term storage. Where it is worth the additional effort, this problem has been overcome by the transformation of these antibody fragments into full-length antibodies. Unfortunately, this conversion is resource intensive and cannot be practically carried out on all selected antibody fragments.

F1 antigen is a capsular protein unique to most *Y. pestis* [4], [5], [6], [7], [8] and therefore a good target for immunological detection of this microorganism. The advantage of using recombinant F1 as a selection target rather than the entire organism is the reduced likelihood of developing antibodies against components of the cell surface that are not associated to pathogenicity. In an attempt to find scFv specifically recognizing F1 antigen we have panned a large phage display library against recombinant F1. In this report we describe selection of a set of seven different αF1 scFv together with their rapid screening and assay for binding specificity by multiplex flow cytometry and one-step ELISA. More importantly we describe phage-based assays in which filamentous phage displaying our selected αF1 scFvs prepared using a helper cell-based method previously described [9] are successfully used as “large antibodies” instead of the soluble scFvs. Phage-scFvs have a number of advantages over the use of free scFvs, including stability, ease of purification and labeling. Furthermore even our weakest αF1 scFv shows binding specificity in a phage display format. Therefore we believe that the use of phage-displayed scFv may be generally applicable to a vast array of scFv, overcoming the typical shortcomings of these antibody fragments and avoiding the need for subcloning.

## Results

### αF1 scFv Antibody Selection, Screening and Identification

**Figure 1:**
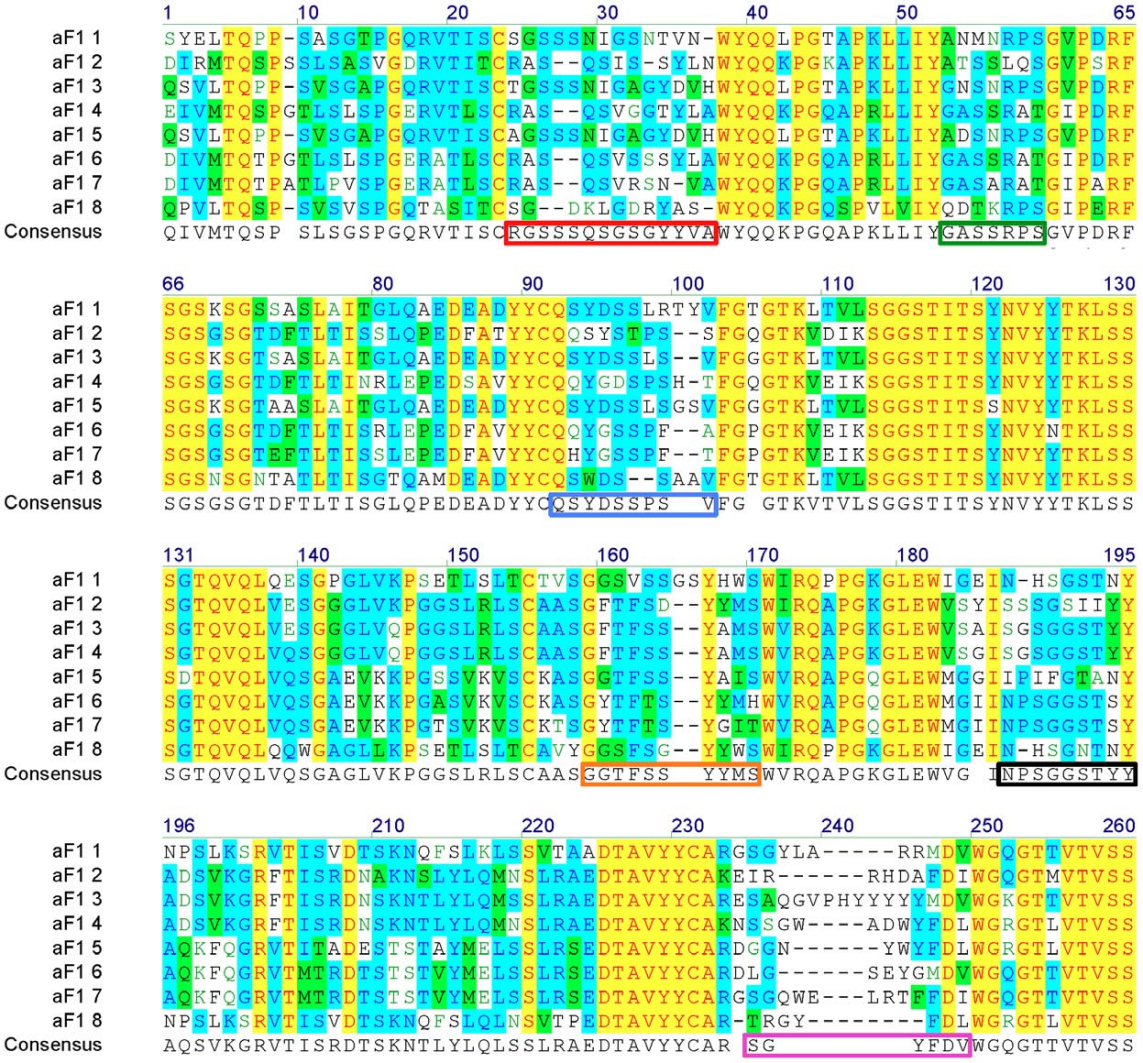
Amino acid sequence alignment of the 8 different αF1 scFv. 36% of the 252 clones that were screened by ELISA specifically bound recombinant F1 antigen. 62 of the 90 positive clones were successfully sequenced. Eight different αF1 scFv groups were identified following alignment of the full-length amino acid sequences obtained by translating the DNA sequences. One clone per group was selected. Here presented are the amino acid sequences of the selected clones. Letters indicated in the following combination of foreground/background: black/white, blue/torquoise, black/green, red/yellow and green/white, correspond to amino acids that are non-similar, conservative, similar, identical or weakly similar respectively. The consensus sequence corresponds to amino acids that are represented at least in 3 of the 8 sequences. The boxed portions of the consensus correspond to the CDRs (Kabat definition). With the red, green, blue, orange, black and pink boxes defining CDRL1 through 3 and CDRH 1 through 3 respectively.

Phage display technology was used to select multiple scFv clones that specifically bound to recombinant F1 antigen. Following library screening, the DNA pool representing the selected scFv population was recovered, and full-length scFv open reading frames were cloned into the pEP-AP vector (Figure S1A). This expresses scFvs as alkaline phosphatase (AP) fusions allowing identification of F1 binders by one-step ELISAs using recombinant F1. Out of 252 clones analyzed, 35% did not express, 27% expressed but did not bind either F1 or the negative control antigen chicken Lysozyme, 2% expressed and reacted with both F1 and chicken Lysozyme. Overall, 36% specifically bound F1 antigen and did not cross react with chicken Lysozyme. DNA sequence analysis identified 8 unique αF1 scFv clones following alignment of full-length amino acid sequences (Figure 1).

### Assay of αF1 scFv Binding to Recombinant F1 Antigen

**Figure 2:**
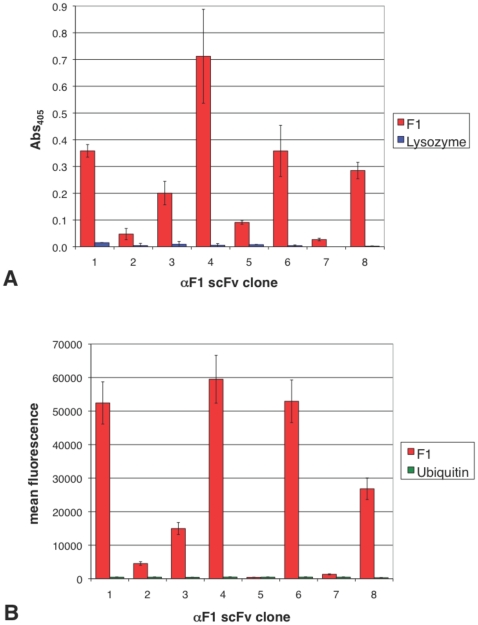
Binding analysis of αF1 scFv to recombinant F1 antigen. **A**) ELISA analysis: The αF1 scFv proteins were expressed as alkaline phosphatase (AP) fusion proteins, recovered from the periplasmic fraction, and tested by one-step ELISA for binding to either recombinant F1 or chicken Lysozyme, without further purification. The binding profile of each αF1 scFv clone presented, was not normalized for expression. **B**) Bead-based flow cytometry analysis: The αF1 scFv proteins were expressed as AP-Ecoil fusion proteins, recovered from the periplasmic fraction and directly labeled with Kcoil-A488. The fluorescently labeled αF1 were tested for binding to recombinant biotinylated F1 and ubiquitin by multiplex bead-based flow cytometry, without further purification. The binding profile of each αF1-APEcoil scFv clone presented was not normalized for expression. The value associated to each bar is an average of three experiments with corresponding standard deviation.

Binding of the 8 selected αF1 scFv antibodies, to recombinant F1 antigen, was confirmed by one-step ELISA as well as multiplex flow cytometry (Figure 2). One-step ELISA demonstrated that all 8 αF1- scFvs bound recombinant F1 antigen, when expressed as AP fusion proteins and did not cross-react with an irrelevant antigen (chicken Lysozyme, Figure 2A). In addition, each αF1 scFv antibody was sub-cloned into a pEP-APEcoil vector (Figure S1B), expressed as AP-Ecoil tag fusion proteins and fluorescently labeled with Kcoil-Alexa Fluor 488 (Kcoil488), through high affinity dimerization with Ecoil peptide tag [10]. Labeled scFv conjugates were directly analyzed by multiplex flow cytometry without purification, adopting a previously described protocol [3]. Analysis was carried out by capturing biotinylated F1 or an irrelevant biotinylated antigen (Ubiquitin) onto 2 different colored luminex bead sets bound to neutravidin. The crude fluorescently labeled αF1-APEcoil fusion proteins were incubated with the microsphere duplex and directly analyzed using a LSRII flow cytometer (Becton Dickinson). Flow cytometry analysis confirmed that 7 of the 8 αF1-APEcoil scFv antibodies bound recombinant F1 and did not cross-react with Ubiquitin (Figure 2B). The overall profile of antibody reactivity when assayed by ELISA or flow cytometry was similar (Figure 2). Generally, αF1 scFv clones 1, 3, 4, 6 and 8 gave the highest binding signals whereas scFv 2 and 7 gave the lowest; scFv clone 5 was weakly positive when analyzed by ELISA and negative by flow cytometry.

### Preparation of multivalent phage-displayed antibodies

Phage antibody libraries are a source of antibody fragments with exquisite binding specificities [3], [11], [12], [13], [14], [15], however, as scFvs, such antibody fragments can be difficult to use, especially if their expression levels and stabilities are low. This proved to be the case with the αF1 antibodies here described, thus limiting their use as detection reagents. Therefore we investigated the possibility of using phage-bound antibodies -i.e. filamentous phage-displayed scFvs- tailoring the subsequent immunoassays to this antibody format. We prepared multivalent phagemid particles displaying either one of our eight αF1 scFvs (CT1 through 8) or anti Lysozyme scFv (CTD1.3) using the helper plasmid system M13cp-CT previously developed in our laboratory [9]. This system has been shown to produce high titers of multivalent phagemid particles. Rapid normalization of CT phage concentrations used in any given experiment was achieved by SDS-PAGE band densitometry based on the intensity of the major protein p8. This way of assessing phage concentrations was preferred to the most commonly used phage titration based on determination of colony forming unit (cfu), in order to avoid artifacts due to multivalent display, namely lower titers caused by reduced phage infectivity [16]. Phage concentrations determined using densitometry versus titration were, as expected, mostly higher (CT3, CT5, CT8, D1.3) and ranged between 3×10^+13^ and 8×10^+11^ as shown in Table S1. The most convenient way to assay the reactivity of our scFv-phage reagents was flow cytometry using fluorescently labeled phagemid particles. We used two labeling methods: in the first bound phage was stained with αM13 primary antibody and PE-conjugated secondary antibody, while in the second phage was directly labeled with fluorescein, prior to binding, using a previously described protocol [17] that resulted in each phagemid particle being labeled with about 50–500 fluorophores (Table S2).

### Assay of αF1-phage scFv Binding to Recombinant F1

**Figure 3:**
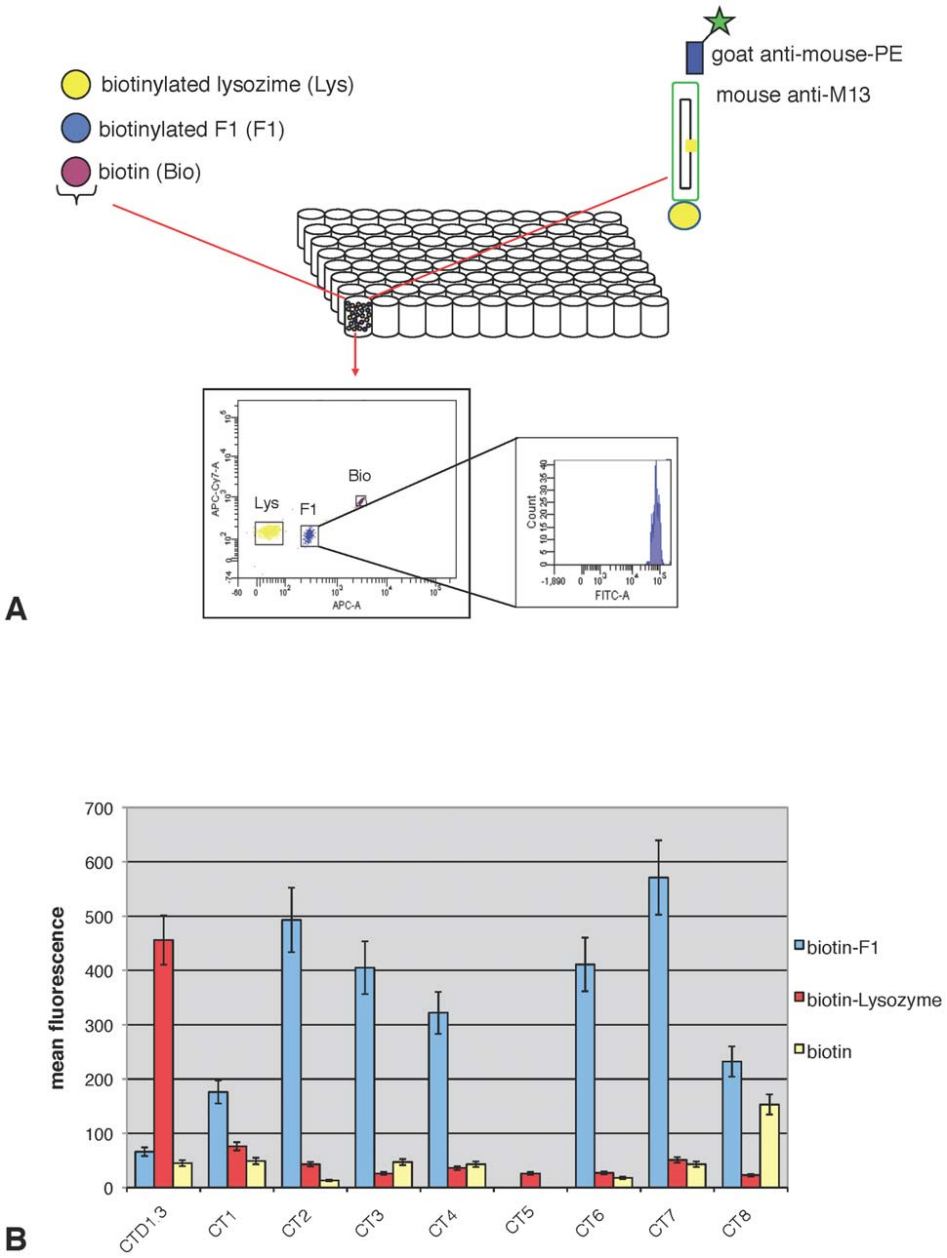
Bead-based flow cytometry analysis: aF1 phage reactivity with recombinant F1 antigen. **A**) Schematic of analysis: a set of 3 distinct luminex beads was bound to biotinylated Lysozyme, biotinylated F1 or biotin respectively. Bound phage was stained with αM13 mouse IgG and phycoerythrin (PE)-conjugated goat αMouse. Beads were separated based on their intrinsic fluorescence (APC-A, APC-cyt7), and the associated PE stain was measured to assess specificity of binding to F1 antigen. **B**) Assay results: Eight different αF1 scFv were expressed in phage format (CT1 through 8). Phage preparations were normalized to a concentration of 5×10^+12^ cfu/mL and analyzed for specific binding. The value associated to each bar is an average of three experiments with corresponding standard deviation.

Reactivity of the αF1 phage-displayed scFv (CT1 through 8) with recombinant F1 was carried out by multiplexed flow cytometry using a microsphere triplex bound to biotin, biotinylated Lysozyme or biotinylated F1, with the first two bead sets acting as negative controls for assessing specificity of F1 binding (Figure 3A). Upon incubation with phage, binding events were reported by staining with αM13 (mouse primary antibody) and PE-conjugated αMouse (secondary antibody). Flow cytometry analysis confirmed that, with the exception of scFv 5, all αF1 scFv antibodies bound recombinant F1 and did not cross-react with Lysozyme (Figure 3B). As expected, the negative control αLysozyme phage-displayed scFv D1.3 (CTD1.3) bound Lysozyme and did not cross-react with F1. Furthermore, with the exception of CT8, none of the scFv-phage interacted with biotin-bound beads. Similar results were obtained with the direct phage labeling method (data not shown), therefore, directly labeled CT1 through 8 were further evaluated as detection reagents for cell-based assay.

### Assay of αF1-phage scFv Binding to *Yersinia* Cells

**Figure 4:**
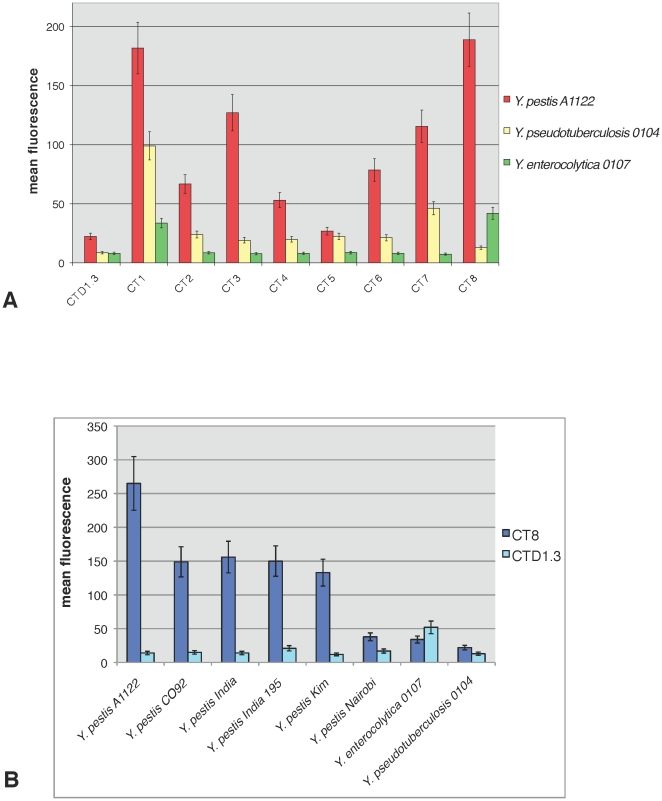
Cell-based flow cytometry analysis: Fluorescent aF1 phage reactivity with fixed *Yersinia* cells. **A**) Reactivity of all aF1 scFv phage clones with 3 fixed *Yersinia* strains: F1-positive *Y. pestis* A1122 and F1-negative strains *Y. enterocolytica* 0107 and *Y. pseudotubeculosis* 0104 were incubated with either αLysozyme (CTD1.3) or αF1 scFv-displaying FITC-labeled phage (CT1 through 8). The fluorescence associated with each cell type was measured using FACS Calibur and data were analyzed by CellQuest. **B**) Reactivity of αF1 CT8 and CTD1.3 with 8 fixed *Yersinia* strains. F1-positive *Y. pestis* strains A1122, C092, India, India 15 and Kim, and F1-negative strains *Y. pestis* Nairobi, *Y. enterocolytica* 0107 and *Y. pseudotuberculosis* 0104 were incubated with FITC-labeled phage. The value associated to each bar is an average of three experiments with corresponding standard deviation.

**Figure 5:**
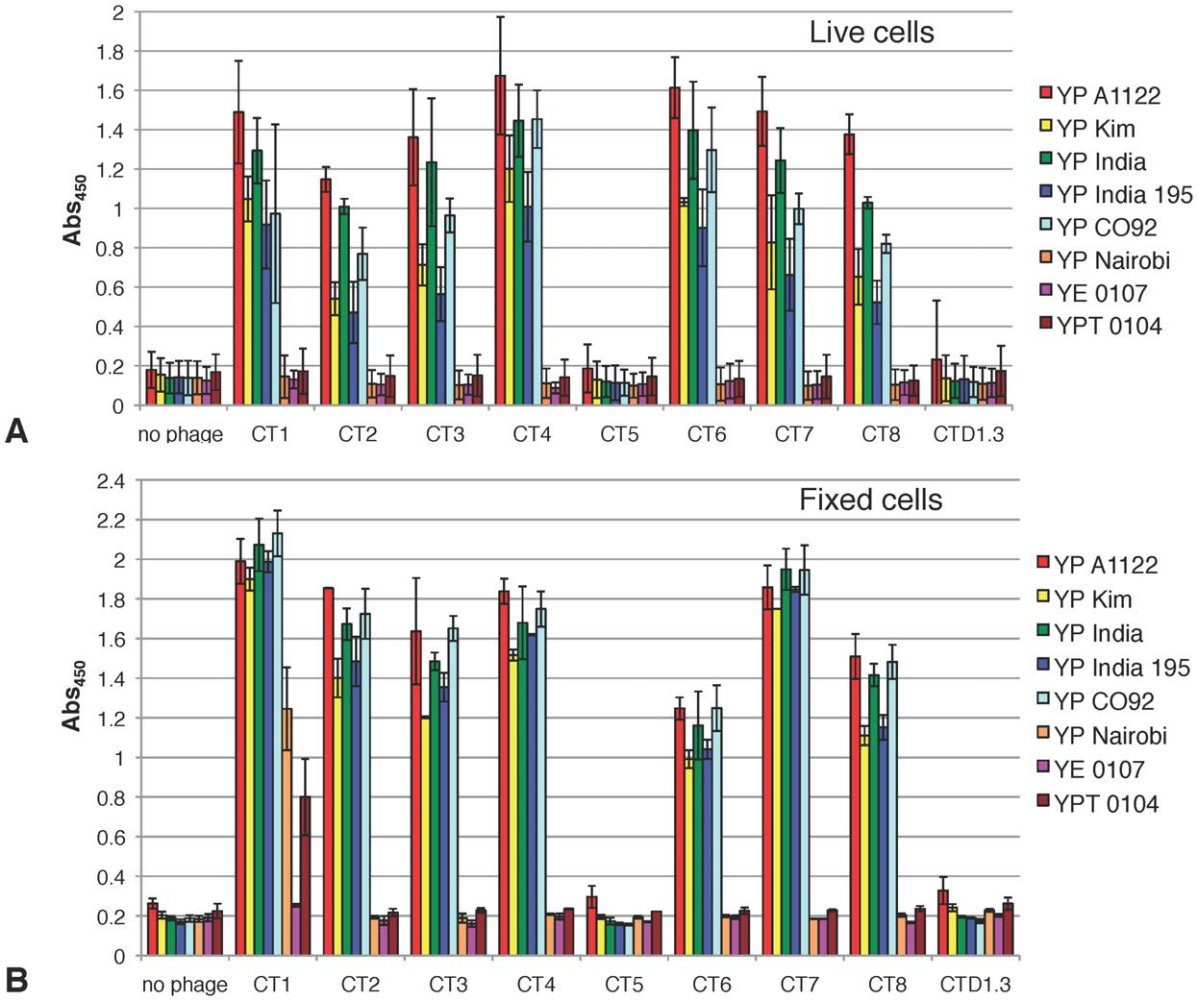
Whole-cell ELISA analysis: aF1 phage reactivity with live or fixed *Yersinia cells*. Each phage-displayed αF1 (CT1 through 8) or αLysozyme (CTD1.3) scFv was incubated with blocked live (**A**) or fixed (**B**) F1-positive *Yersinia pestis* (YP) A1122, Kim, India and India 195 or F1-negative YP Nairobi, *Yersinia pseudotuberculosis* 0104 (YPT 0104) and *Yersinia enterocolytica* 0107 (YE 0107). Phage-binding events were reported using αM13-HRP antibody. Background noise coming from buffers, secondary antibody or the cells was evaluated by including wells with no added phage (no phage). The value associated to each bar is an average of three experiments with corresponding standard deviation.

All our αF1 phage clones were tested for binding to fixed *Y. pestis* cells by flow cytometry (Figure 4) using CTD1.3 as negative control. Assays were performed using equivalent amounts of labeled phage. Average FITC labeling was 90 molecules of dye per phage particle. All CT phages were initially tested for reactivity with F1 positive *Y. pestis* A1122, and F1 negative strains *Y. pseudotuberculosis* 0104 and *Y. enterocolytica* (Figure 4A). Significant increments above CTD1.3 background binding were detected for all αF1 CT phages with the exception of CT5, which was confirmed to be inactive in accordance with previous results (Figures 2 and 3). CT8 was identified as the phage with the strongest binding to F1 positive strain *Y. pestis* A1122 and the highest increment in fluorescent signal, over the negative control CTD1.3 phage. Therefore CT8 was subsequently tested against a panel of five F1-positive *Y. pestis* strains along with 3 F1-negative *Yersinia* strains including *Y. pestis* Nairobi (Figure 4B). Results show that the interaction of labeled CT8 with F1-positive *Y. pestis* strains was between 3 and 8-fold stronger than binding to the most highly reactive negative cell line, *Y. pestis* Nairobi. Since the cell-based assays performed this far utilized fixed cells we wanted to exclude eventual artifacts due to cell fixation by comparing the reactivity of αF1 phage with fixed versus live *Yersinia* strains (Figure 5). To this end, ELISA was the assay of choice, despite it's impracticality, since the involvement of live cells made it necessary to conduct the test in a containment level 3 laboratory where flow cytometry resources were not available. Phage was incubated with blocked *Yersinia* cells immobilized on plastic using *Y. pestis* (YP) Nairobi, *Y. pseudotuberculosis* 0104 (YPT 0104) and *Y. enterocolytica* (YE) as F1-negative controls. Phage binding events were reported using horseradish peroxidase-conjugated anti M13 antibody (αM13-HRP). Background noise coming from either the buffers or the cells adhering to the wells was evaluated by including wells with no added phage (“no phage” in Figure 5). Non-specific phage interaction with *Yersinia* cells was evaluated using negative control phage CTD1.3. The absorbance values corresponding to this control and the “no phage” control were negligible. Once again, specificity of interaction was determined by comparing binding of CT1-8 to F1-positive versus F1-negative strains. All active αF1 phages bound more strongly to the F1-positive cell lines than to the mostly highly cross-reacting F1-negative strain YPT 0104 with a maximum of 12-fold for CT4 binding to live YP A1122 and a minimum of 2.5-fold for CT1 binding to fixed YP Kim. Whether using live or fixed cells, the trend of binding specificity was similar for all αF1 phage except for CT1 that exhibited unusually high cross-reactivity with fixed F1-negative YP Nairobi and YPT 0104 but not with their live counterpart.

### αF1-phage scFv stability test

**Figure 6:**
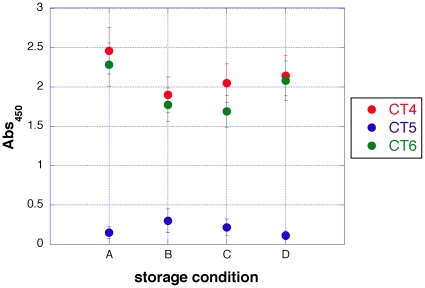
aF1 CT phage are stable, and reactive, following prolonged storage. Phage-displayed αF1 scFv CT4, CT6 and CT5 (inactive control) were treated with a preservative solution and tested for reactivity following different storage conditions; A) freshly prepared phage, B) 6 months at 4°C, C) 9 months at 4°C followed by 1 month at room temperature (RT) and D) 9 months at 4°C followed by 2 months at RT. Phage was tested for activity at non-saturating concentrations by whole-cell ELISA using live *Yersinia pestis* A1122 cells. Each value is an average of 3 experiments with corresponding standard deviation.

In order to further evaluate the usefulness of the αF1-CT phage as detection reagents, the stability of the phage preparations was determined (Figure 6). Three different phage-displayed αF1 scFv antibodies, including 1 negative (CT5) and 2 positive (CT4 and CT5) clones, were tested by ELISA for binding to live YP A1122 cells over a period of 9 months, using different storage conditions. Results show that the phage preparations can be stored for up to 9 months at 4°C, followed by 2 months at room temperature, without significant loss of activity.

## Discussion

The initial aim of this study was to identify scFv antibodies that could be used for *Y. pestis* immunodetection using a recombinant form of one of the most common and species-specific surface antigens of this organism, F1 [8], as a selection target. scFvs were selected from a previously described large scFv phage display library [18] using recombinant F1 as the selection target. 252 clones from the third cycle of library panning were screened after recloning the scFvs as alkaline phosphatase (AP)-fused proteins. Eight distinct clones were identified, further analysis of which was greatly facilitated by expressing the scFv-AP fusion proteins with an additional Ecoil peptide tag without loss of reactivity (Figure 2). In this way crude proteins could be fluorescently labeled using Kcoil peptides labeled with Alexa Fluor 488, in a one-step binding protocol, without any further purification. By taking advantage of the highly specific, high affinity Kcoil-Ecoil interaction [19] labeled scFvs can be easily analyzed by high-throughput multiplex flow cytometry [3]. This allowed us to confirm binding to recombinant F1 and lack of cross reactivity to the negative antigen by multiplex flow cytometry (Figure 2B), with very similar binding profiles to those we obtained by ELISA (Figure 2A). This confirms that when analyzing affinity reagents, multiplex flow cytometry can be used as a rapid, more convenient (high data quantity and low antigen consumption) alternative assay to ELISA.

Although we were able to carry out screening on crude proteins, unfortunately, further characterization and potential development of purified αF1 scFvs as detection reagents, was limited by poor yields, insufficient purity and instability. While particularly valuable antibodies have been converted to full length IgG [12], this procedure is relatively labor intensive and not suitable for all our selected scFvs. In order to overcome this hurdle we investigated the possibility of using phage antibodies themselves – i.e. filamentous phage displaying scFvs - as detection reagents. This has been previously reported [17], [20], but not widely adopted. In part, perhaps, because of the relatively low display levels achievable when using a phagemid system: it is estimated that only 1% of phagemid particles actually display, and these display only a single antibody fragment [21]. While this could have been overcome by recloning each of the scFvs into a phage display vector, we recently described a multivalent display system that can be used to display scFvs in a multivalent format from phagemid vectors without the need for subcloning [9]. In particular, the M13cp-CT helper plasmid, in which p3 is truncated, showed the strongest ELISA signals when compared to standard phagemid display, or display using helper plasmids in which p3 was either full length or completely deleted [9]. We were able to successfully assay multivalent phage displaying αF1 (CT1 through 8) and αLysozyme scFvs (CTD1.3) for binding to their respective antigens by multiplex flow cytometry using phage labeled with mouse αM13 IgG and phycoerythrin (PE)-conjugated goat anti-mouse as immunochemical reagents (Figure 3). This assay confirmed that when converted to phage format, 7 of the 8 scFvs specifically interacted with F1 (scFv 5 was inactive).

We further studied the binding of phage-displayed scFv to *Y. pestis* by whole-cell ELISA (Figure 5) and by flow cytometry (Figure 4). In the former assay we used live and fixed cells, which revealed that the use of fixed *Y. pestis* may lead to artifacts. Furthermore, the live cell-binding profile was consistent between all 7 αF1 phage-antibodies tested, with *Y. pestis* A1122 binding the most, and *Y. pestis* India 195 binding the least (Figure 5A), suggesting that all the scFvs may target the same F1 epitope. In the flow cytometry assay we used directly labeled fluorescent phage, which allowed us to streamline the protocol and reduce it to only two simple binding and washing steps. The phage labeling procedure was straightforward and resulted in an average of 250 fluorophore molecules per phage particle (Table S2). This level of labeling is much higher than levels desirable when phage-free proteins are labeled directly. In part this is due to the deleterious effects of excessive labeling on protein function, which is less of a problem when phage are directly labeled, as most of the label is attached to the major coat protein p8 [17] and therefore less likely to interfere with the function of the displayed scFv. Figures 3, 4 and 5 show the versatility of phage-displayed scFvs as demonstrated by their successful use in a wide variety of assays. In the cell-binding experiments (Figures 4 and 5), phage clearly bound to those *Y. pestis* strains known to express F1, and did not bind to the F1-negative strains. In the experiments testing binding to recombinant F1 the signal to noise ratios obtained using phage-displayed scFv (Figure 3B) are slightly lower than those obtained with phage-free proteins (Figure 2B). However the specificity of binding is clear in both cases, furthermore a slight reduction in sensitivity is a price well worth to pay since phage-displayed scFvs are far easier to purify/label and more stable than their self-standing counterpart.

To our great surprise, prolonged storage of phage antibodies at room temperature and/or 4°C did not compromise activity (Figure 6). While phage infecting ability under extreme conditions is known to be very stable [22], it is interesting that the binding activity of displayed proteins also appears to be intact after many months of storage. This suggests that appropriately designed assays using well-characterized phage antibodies could be very useful as reagents in suboptimal conditions. The use of phage antibodies as immunochemical reagents has been previously reported for western blotting and immunohistochemistry [20], [23]. The work reported here augments the number of assays for which phage antibodies can be used to include flow cytometry.

In conclusion we have selected and characterized a set of scFv that specifically bind recombinant F1 antigen. We have also demonstrated that phage-displayed αF1 scFv are robust, effective and reliable reagents in immunoassays designed to detect the presence of live or fixed F1-positive *Yersinia pestis*. Furthermore, based on our results, we feel confident to generally state that phage-displayed proteins might be more user-friendly reagents than self-standing proteins, due to ease of purification, efficiency and convenience of labeling protocols and exceptional stability.

## Materials and Methods

### Selection of αF1 scFv Antibodies by Phage Display

#### Preparation of Biotinylated F1 Antigen

The recombinant F1 antigen was provided by DSTL labs (Porton Down, Salisbury, Wiltshire). Purified F1 protein was biotinylated using EZ-Link® NHS-LC-LC Biotin (Pierce, 21343) according to the manufacturer's instructions. The level of biotinylation was quantified as 3–5 molecules of biotin per mole of protein, using the EZ™ Biotin Quantification Kit (Pierce, 28005).

#### Panning

The scFv phage library has been previously described [18]. The selection procedure was automated using the Kingfisher magnetic bead system (Thermo Lab Systems) and allowed panning to be carried out in-solution. Prior to selection, 1×10^+12^ colony forming units (cfu) of scFv phage library were blocked using 2% BSA PBS + 0.01% Tween 20 (PBS-LT), at room temperature (RT) for 1 h in a final volume of 134 µL. In the first selection round, 1.8 µg of biotinylated F1 antigen was incubated with the blocked scFv library at room temperature (RT) for 1 h, in a final volume of 190 µL. Following incubation, the scFv phage-F1 complexes were captured onto 2×10^+7^ streptavidin magnetic beads (Dynabeads, M-280, Miltenyi Biotech, 112.05D) by incubation at RT for 15 min. The bead complex was then washed 3 times in 190 µL of PBS-LT and 3 times with 190 µL of PBS + 0.1% Tween 20 (PBS-T). The final scFv phage binding population was eluted via incubation at RT for 3 min and 30 s using 180 µL of 0.1 M glycine pH 2.2, and neutralized to pH 7.5 by the addition of 50 µL 1 M Tris-HCl (pH 8.8). The eluted scFv phage population was recovered by infecting 100 µL into 1 mL of *Escherichia coli* DH5αF' cells at OD_600_  =  0.5, by static incubation at 37°C for 45 minutes. The phage-infected bacterial cells were plated onto 2XYT agar containing 100 µg/mL carbenicillin and 3% Glucose (2XYT/Carb/Glu) and incubated overnight (O/N) at 30°C. The phage-infected bacterial cells were recovered in 2 mL of 2XYT/Carb/Glu broth, 10 µL of the bacterial suspension was inoculated into 10 mL of 2XYT/Carb/Glu broth and incubated with shaking (260 rpm) at 37°C until the OD_600_ was 0.5. The cells were subsequently co-infected with 1.3×10^+13^ cfu of M13K07 helper phage (Amersham Pharmacia) by static incubation for 30 min at 37°C. Following infection, the cells were recovered by centrifugation at 3000 rpm for 30 min and re-suspended in 10 mL of 2XYT broth containing 100 µg/mL carbenicillin, 25 µg/mL kanamycin and incubated O/N at 30°C with shaking (260 rpm). The amplified scFv phage were recovered within the media supernatant by centrifugation at 3000 rpm for 30 min. 171 µL of the supernatant was used directly in the subsequent selection round. The amplified phage particles were precipitated from the remainder of the media supernatant by two rounds of PEG/NaCl precipitation and stored at 4°C. The stringency of the selection conditions was increased through 3 subsequent rounds of panning. The biotinylated F1 antigen concentration was decreased 10 fold (from 600 nM to 60 nM) from the first to second, and third round and wash times were increased (1, 5 and 15 min) with each subsequent round.

### Screening of Selected αF1 scFv Antibodies

#### Expression of scFv antibodies as Alkaline Phosphatase Fusion Proteins

The DNA encoding the scFv antibodies, produced in the αF1 third round selection output, was recovered from 100 µL of the infected bacterial cells, using the QIAprep spin miniprep kit (Qiagen, 27104). The DNA, encoding full-length scFv antibodies, was recovered by digestion with NEB restriction enzymes *BssH*II and *Nhe*I, purified using QIAquick gel extraction kit (Qiagen, 28704) and ligated into *BssH*II and *Nhe*I digested pEP-AP vector (Figure S1A). 1 µL of the ligation reaction was transformed into 50 µL of BL21 Gold DE3 electrocompetent bacterial cells (Stratagene, 230132) and plated on kanamycin (50 µg/mL) agar. Approximately 250 scFv clones were picked using QBot (Genetix) and inoculated into 1 mL of kanamycin selective (50 µg/mL) auto-induction media [24] in a 96 deep well plate (Thomson Instrument Co., 951652). Following inoculation, the plate was incubated with shaking at 18°C for 36 h. The cells were recovered by centrifugation at 3,000 rpm for 30 min; the supernatant was discarded and 300 µL of Popculture (Invitrogen, 71092) was added to the pellet (0.2-fold final concentration) and incubated at RT for 15 min with shaking (900 rpm). 1 mL of PBS was added to each well and the plate was centrifuged at 4,000 rpm for 30 min to pellet bacterial debris. The supernatant containing the scFv protein was transferred into a fresh 96 well deep well plate and stored at 4°C.

#### One-Step ELISA

The expressed αF1-AP scFv fusion proteins were analyzed by one-step ELISA for binding to recombinant F1 antigen, or cross reactivity to chicken Lysozyme. The ELISA was automated using the liquid handling Genesis 2000 workstation (Tecan). 192 wells of a 384 Maxisorp plate (NUNC #464718) were coated with 0.1 µg of F1 antigen and all remaining wells were pre-coated with 0.1 µg of chicken Lysozyme (Sigma, L7651), allowing duplicate analysis of 96 different scFv clones against both antigens. The plate was incubated O/N at 4°C and the following day, unbound antigen was removed, the plate washed twice with PBS-LT and blocked with 100 µL of 1% w/v BSA (Sigma, A7906), by incubation for 1 h at RT. The block buffer was removed and the plate washed twice with PBS-LT. 50 µL of scFv antibody periplasmic extract was added, in duplicate, to wells containing either Lysozyme or F1 antigen and incubated at RT for 1 h. The plate was washed 3 times with PBS-T and 3 times with PBS-LT. Binding events were detected following addition of 80 µL of Alkaline Phosphatase (AP) substrate *p*-Nitrophenyl Phosphate Disodium Salt (PNPP, Pierce, 37620). In a second Maxisorp 96 well plate, individual scFv expression levels were determined by addition of 10 µL of scFv antibody periplasmic extract to 80 µL of AP substrate. Immediately following addition of AP substrate, the absorbance at 405 nm was measured using the SPECTRA fluor Plus spectrofluorometer. The time zero value was deducted from subsequent absorbance values generated as the AP signal increased over time.

#### Sequencing of αF1 scFv

62 clones, identified as positively binding to F1 antigen by ELISA, were sequenced (Ohio State University Plant-Microbe Genomics Facility) using forward primer T7 Promoter (5′ TAATACGACTCACTATA 3′) and reverse primer APRev (5′ CAGGTTTATCGCTAAGAGAAT 3′). The resulting DNA sequence was analyzed using Vector NTI Advance 10 ContigExpress (Invitrogen) and translated, and aligned, using Vector NTI Advance 10 Alignx (Invitrogen) to identify different scFv groups.

#### Generation and Multiplex Flow Cyometry Analysis of scFv-APEcoil

The DNA encoding 8 different scFv antibodies was sub-cloned from the pEP-AP vector into pEP-AP-Ecoil vector (Figure S1B) by digestion with NEB restriction enzymes *BssH*II and *Nhe*I as described previously. Clones were sequenced with both T7 Promoter (5′ TAATACGACTCACTATA 3′) and APRev (5′ CAGGTTTATCGCTAAGAGAAT 3′). The eight different αF1 scFv antibodies were expressed in BL21 Gold DE3 (Stratagene, 230132), as described earlier, and analyzed by multiplex flow cytometry as reported in [3]. Two different color-coded, carboxylated microsphere suspension (Luminex®, xMAP™ 136 and 142), were coupled with neutravidin and coated with either biotinylated F1 (bead set 136) or biotinylated Ubiquitin (bead set 142). The antigen-coupled microspheres were combined to create a duplex that was incubated with crude aF1 scFv labeled with Alexa Fluor 488-labelled Kcoil [19] (KcoilA488) and directly analyzed by flow cytometry. The mean fluorescence data of each bead set, within the multiplex, was collected using the high-throughput analysis feature of the Becton Dickinson LSRII flow cytometer and analyzed by DIVA software. The bead duplex was separated into gates by excitation using a 633 nm laser and emission detection through 780/60BP and 660/20BP filters. The mean fluorescent value of each gate was recorded following excitation using the 488 nm laser and emission detection through a 530/80BP filter. 125 µL each sample was injected at a rate of 0.5 µL per sec and the mean fluorescence of 3000 gated, microspheres was recorded.

### Production and Labeling of Phage-displayed αF1 scFv

#### scFv Cloning in Phagemid Vector

*BssH*II and *Nhe*I digested DNA fragments encoding each αF1 scFv antibody (described earlier) were ligated into *BssH*II and *Nhe*I digested pDAN5 [18]. Ligase mixtures were transformed into chemically competent *E. coli* DH5aF'. Transformants were plated on 2XYT/Carb/Glu agar. Two colonies were picked for each αF1 scFv, inoculated onto 2XYT/Carb/Glu broth and incubated O/N at 37°C. The DNA from each clone was recovered using the QIAprep spin miniprep kit (Qiagen) according to the manufacturer's instructions. Plasmids were used as template for amplification of scFv genes with primers PDPH3′ (5′ TAACGTCTGGAAAGACGACAA 3′) and PDPH5′ (5′GCAGCCGCTGGATTGTTATTA 3′). A PCR master mix was prepared containing 395 µL Milli-Q autoclaved water, 50 µL Taq thermo buffer (NEB), 20 µL dNTP (NEB, 10 µM each), 10 µL of 10 µM PDPH3′ primer, 10 µL of 10 µM PDPH5′ primer and 5 µL (25 units) of Taq Polymerase (NEB). 19.6 µL master mix was mixed with 0.4 µL plasmid miniprep and amplification occurred under the following conditions: 94°C for 5 min, (95°C for 45 sec, 55°C for 45 sec, 72°C for 1 min) ×40 cycles, 72°C for 7 min. The identity of the clones was confirmed by size, DNA fingerprinting (digestion with *BstN*I restriction enzyme [25]) and sequencing.

#### Phage production

DNA plasmids, encoding each selected αF1 and αLysozyme (negative control) scFv were transformed into M13cp-CT helper cells [9]. The resulting single clone transformants were used to produce phage (CT1 through 8 and CTD1.3) according to the following protocol. O/N cultures of the M13cp-CT helper cell transformants were obtained in 2 mL 2XYT supplemented with carbenicillin and chloroamphenicol (50 and 20 µg/mL respectively, 2XYT/Carb/Cap). 100 µL of O/N culture was inoculated into 10 mL of 2XYT/Carb/Cap broth and incubated at 37°C to OD_600_  =  0.5. Cells were centrifuged at 4,000 rpm for 15 min and cell pellets were re-suspended in 50 mL 2XYT supplemented with carbenicillin (25 µg/mL final concentration, 2XYT-Carb). Following O/N incubation at 30°C with shaking (260 rpm) the cultures were centrifuged at 4,000 rpm for 30 min. The supernatant containing phage was recovered and the phage was PEG/NaCl precipitated at 4°C O/N. Phage precipitates were recovered by centrifugation at 10,000 rpm for 30 min. The resulting phage pellet was re-suspended in 15 mL PBS and the suspension was cleared by centrifugation at 7,000 rpm for 15 min. The phage sample was PEG/NaCl precipitated on ice for 1 h. Precipitated phage was recovered following centrifugation at 10,000 rpm for 30 min, phage pellets were resuspended in 1 mL PBS, cleared by centrifugation and stored at 4°C. Phage was amplified from the phage stocks according to the following method. M13cp-CT helper cells overnight cultures were diluted 1∶50 into 10 mL of 2XYT-Cap broth and incubated at 37°C to OD_600_  =  0.5. Each phage stock was diluted in 2XYT medium to a final concentration of 1.8×10^+9^ cfu/mL. 1.8E+9 cfu of each phage was added to 10 mL of M13cp-CT cell (1∶1 multiplicity of infection). Infection occurred at 37°C for 30 min, cells were harvested by centrifugation and resuspended in 50 mL 2XYT-Carb. Additional steps are as for previously described phage production protocol. Phage concentration was determined by either titration (infection of DH5αF' with serial dilution of phage and plating onto LB-Carb agar) or densitometry. For the latter method 10-fold diluted phage solutions were analyzed by SDS-PAGE together with serial dilution of wild type phage (M13K07) of known titer. The p8 band intensity of scFv-phage was translated into concentrations using the equation defining the standard curve obtained by plotting known M13K07 concentrations (determined by titration) versus corresponding p8 band intensities.

#### Phage Labeling

CT1-8, CTD1.3 and wt phage was labeled according to a slight modification of a previously described procedure [17]. Phage preparations were PEG/NaCl precipitated and re-suspended in bicarbonate buffer pH 9 (1 M, conjugation buffer,). Fluorescein isothiocyanate (FITC, Sigma, F3651) was added to a final concentration of 0.2 mg/mL from a 5 mg/mL stock solution in conjugation buffer. Mixtures were incubated at RT with rotation in the dark. Labeled phage were purified by three cycles of PEG/NaCl precipitation and re-suspended in PBS. The FITC concentration in each phage preparation was determined by UV/vis absorbance (Abs_494_/71000) using unlabelled phage as blank. The degree of labeling was determined by dividing FITC concentration by phage concentration (both expressed as particles/volume), resulting in a level of labeling ranging between 30 and 150 fluorophore molecules per phagemid particle.

### Assay of Phage-displayed aF1 scFv

#### Multiplex Flow Cyometry Assay of Binding to Recombinant F1

The multiplex flow cytometry assay was performed according to a slight modification of a previously described method [3]. Three different color-coded, carboxylated microsphere (Luminex®, xMAP™), were coupled with neutravidin and coated with either biotinylated F1, biotinylated Lysozyme (for assessment of binding specificity) or with biotin (no antigen, for assessment of bead-binding level). Equal volumes of the antigen-coupled microsphere suspensions were combined to create a triplex (8 µL), that was incubated with 100 uL phage (4×10^+12^ phage/mL determined by the densitometry-based method described above) at RT for 1 h with mixing. Upon washing the beads were re-suspend in 100 µl of 10-fold diluted wonderblock (WB, PBS + 0.3% BSA, 0.3% fish gelatin, 0.3% milk, WB) in PBS and 1∶500 diluted mouse αM13 (ARP, 03-65197) following incubation at RT for 1 h with shaking. After washing, phage-bound beads were re-suspended in 100 µl of 10-fold diluted WB in PBS and 1∶1000 diluted goat anti-mouse-PE (Molecular Probes, P21129). After more washing the beads were re-suspended in 200 µl of PBS and analyzed by flow cytometry as described above.

#### Yersinia Cell Preparation

All *Y. pestis* strains are described in [26], and were originally obtained from the Reference Collection of the Centers for Disease Control and Prevention, Fort Collins, CO. Y. *pestis* A1122, Kim, India, India 195, CO92 are F1-positive strains while *Y. pestis* Nairobi, *Y. enterocolytica* 0107 and *Y. pseudotuberculosis* 0104 are F1-negative strains. 1 µl of cell glycerol stock was inoculated into 5 ml TSB (tryptone soya broth) medium in a 50 mL falcon tube. The culture was incubated at the 28°C with moderate shaking (240 rpm) for 15–16 hours. Live cells were directly re-suspended in PBS and used in the experimental assay. Whereas those cells to be fixed were washed 3 times with PBS and re-suspended in 4% paraformaldehyde. Cell death was confirmed by culturing paraformaldehyde treated cells on TSB agar plates for 2 days at 37°C.

#### Flow Cytometry Assay of Binding to Yersinia Cells

Fixed cells were blocked by re-suspension in WB to a final OD_600_ of 0.1 and incubated either at 4°C O/N or at RT for 1 h. Cells were harvested by centrifugation (10 min, 4000 rpm) and re-suspended in a volume of PBS 10-fold lower than the volume of blocking solution. 1 µL of blocked cells (about 1×10^+6^ cells) were incubated with 8 µL of FITC-labeled CT1-8 (6×10^+11^ cfu/mL) or 8 µL negative control phage (wt or CTD1.3 4×10^+12^ cfu/mL) in 5-fold diluted WB. Cells were incubated at RT for 2.5 h, washed twice with PBS and analyzed by flow cytometry. The fluorescence intensity of each cell type was measured on a FACSCalibur (Becton Dickinson, Franklin Lakes, NJ) flow cytometer with laser excitation at 488 nm and fluorescence emission collection through a 530/30 nm filter. Untreated cells were also analyzed to assess auto fluorescence, which was negligible. Resultant data were analyzed by CellQuest software.

#### ELISA of binding to Yersinia cells

The ELISA was performed in triplicate under Containment Level 3 Biosafety conditions. 100 µL live or fixed *Yersinia* cells (in PBS OD_600_  =  0.2) were transferred into each well of 96-well flat bottom Nunc Maxisorp plate (Thermo Scientific, 449824). The plate was incubated O/N at 4°C. Unattached cells were aspirated and plate was washed once with PBS + 0.05% Tween 20 (wash buffer). All the washes were performed by aspiration to eliminate the creation of aerosols. The plate was blocked by addition of 250 µl WB for 1 h at 37°C. scFv-phage (CT1-8 or CTD1.3 as a negative control) was diluted to 4.5×10^+9^ cfu/mL (determined by densitometry) in WB. 60 µL of each phage solution was added to each well and the plate was incubated at RT with gentle agitation for 2 h and then washed four times with wash buffer and additional four times with PBS + 0.005% Tween 20. aM13-HRP antibody conjugate (GE Healthcare, 27-9421-01) was diluted 1∶1000 into 10-fold diluted WB and 60 µL was added to each well of the plate. Following incubation at RT for 1 h, with shaking, the plate was washed as described previously. 100 µL of TMB substrate (Pierce, 34028) was added to each well and the reaction was stopped by the addition of 50 µL 1 M H_2_SO_4_. The absorbance was read at 450 nm using victor3 plate reader (PerkinElmer).

#### Phage Stability Test

CT4 CT6 and CT5 (negative control) were solubilized in WB, protease inhibitor cocktail (Roche, 05892791001) and NaN_3_ (0.02% final concentration). Phage clones were stored in various conditions including 4°C for 6 months; 4°C for 9 months followed by room temperature for 1 month; 4°C for 9 months followed by room temperature for 2 months. These phage samples and their freshly prepared counterpart were tested by phage ELISA using live *Y. pestis* A1122 as described above, in triplicate, using a fixed concentration of phage (4.5×10^+9^ cfu/mL).

## Supporting Information

Figure S1 **Genetic maps of pEP-AP (A) and pEP-APEcoil (B) vectors.**
(TIFF)

Table S1 **A typical set of phage concentrations: Concentration of phage displaying αF1 (CT1-8) or αLysozyme (CTD1.3) scFv were obtained by standard titration and by densitometry.** Each value corresponds to the average of two experiments.
(DOCX)

Table S2 **A typical set of phage labeling efficiencies: Upon labeling, concentration of phage displaying αF1 or αLysozyme scFv (CT1-8 and CTD1.3 respectively) were determined by densitometry and concentration of FITC was determined by absorbance at 494 nm.** Ratio of FITC to phage concentration allowed determination of labeling efficiency. Each value corresponds to the average of two experiments.
(DOCX)
