# Correction: Development of Phage-Based Single Chain Fv Antibody Reagents for Detection of *Yersinia pestis*

**DOI:** 10.1371/annotation/fd1d120c-ff3d-4873-85e1-ee70bf010241

**Published:** 2012-04-18

**Authors:** Antonietta M. Lillo, Joanne E. Ayriss, Yulin Shou, Steven W. Graves, Andrew R. M. Bradbury, Peter Pavlik

There was an error in the by-line in the online version of the article. The sixth author, Peter Pavlik, was mistakenly excluded. 

