# Supplementary figures and images for: Development of Phage-Based Single Chain Fv Antibody Reagents for Detection of Yersinia pestis
Source: PLoS One. 2011 Dec 8;6(12):e27756. doi: 10.1371/journal.pone.0027756 (PMC3234238; doi:10.1371/journal.pone.0027756)

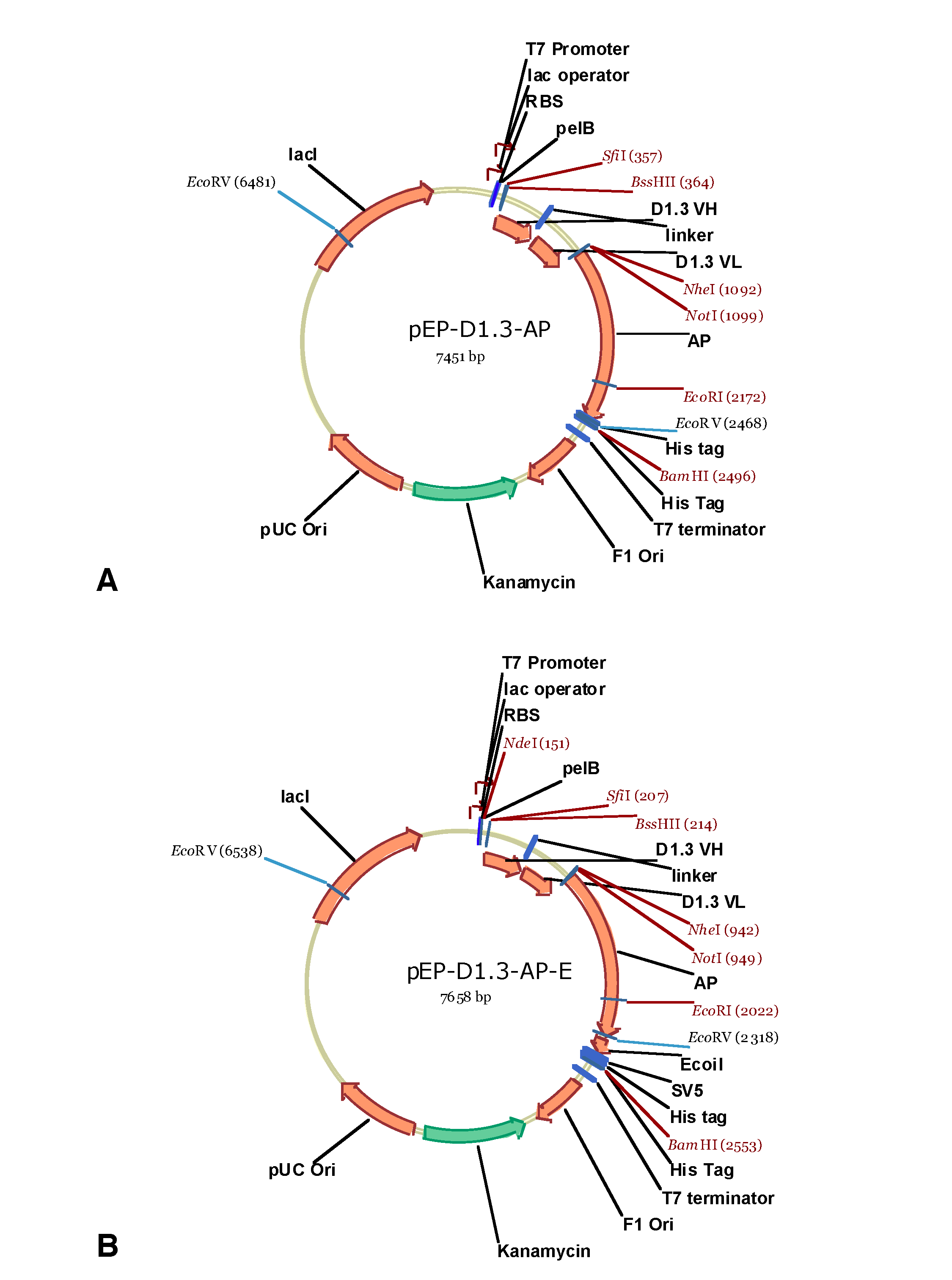

Supplement: Figure S1 — Genetic maps of pEP-AP (A) and pEP-APEcoil (B) vectors. (TIFF) [file pone.0027756.s001.tiff]
