# Supplementary material for: Development of Phage-Based Single Chain Fv Antibody Reagents for Detection of Yersinia pestis
Source: PLoS One. 2011 Dec 8;6(12):e27756. doi: 10.1371/journal.pone.0027756 (PMC3234238; doi:10.1371/journal.pone.0027756)
Supplement: Table S1 — A typical set of phage concentrations: Concentration of phage displaying αF1 (CT1-8) or αLysozyme (CTD1.3) scFv were obtained by standard titration and by densitometry. Each value corresponds to the average of two experiments. (DOCX) [file pone.0027756.s002.docx]

|  | **[phage] by densitometry**  (cfu/mL) | **[phage] by titer**  (cfu/mL) |
| --- | --- | --- |
| CT1 | 2.00E+12 | 2.50E+12 |
| CT2 | 2.1E+13 | 2.08E+13 |
| CT3 | 2.8E+13 | 1.85E+13 |
| CT4 | 3.4E+13 | 2.35E+13 |
| CT5 | 2.9E+13 | 1.81E+13 |
| CT6 | 2.4E+13 | 2.10E+13 |
| CT7 | 1.9E+13 | 1.75E+13 |
| CT8 | 9.2E+12 | 7.62E+12 |
| CTD1.3 | 3.2E+13 | 1.78E+13 |
