# Supplementary material for: Development of Phage-Based Single Chain Fv Antibody Reagents for Detection of Yersinia pestis
Source: PLoS One. 2011 Dec 8;6(12):e27756. doi: 10.1371/journal.pone.0027756 (PMC3234238; doi:10.1371/journal.pone.0027756)
Supplement: Table S2 — A typical set of phage labeling efficiencies: Upon labeling, concentration of phage displaying αF1 or αLysozyme scFv (CT1-8 and CTD1.3 respectively) were determined by densitometry and concentration of FITC was determined by absorbance at 494 nm. Ratio of FITC to phage concentration allowed determination of labeling efficiency. Each value corresponds to the average of two experiments. (DOCX) [file pone.0027756.s003.docx]

|  | **[phage]**^a^  (cfu/mL ) | **Abs_494_**^b^ | **[FITC]**^c^  (M) | **[FITC]**^d^  (molecules/mL) | **labeling efficiency**  (FITC /phage) |
| --- | --- | --- | --- | --- | --- |
| CT1 | 2.0E+12 | 1.3E-01 | 1.9E-06 | 1.1E+15 | 5.6E+02 |
| CT2 | 3.6E+12 | 6.4E-02 | 9.1E-07 | 5.4E+14 | 1.5E+02 |
| CT3 | 3.8E+12 | 4.8E-02 | 6.8E-07 | 4.1E+14 | 1.1E+02 |
| CT4 | 3.4E+12 | 3.5E-02 | 4.9E-07 | 2.9E+14 | 8.7E+01 |
| CT5 | 3.8E+12 | 3.5E-02 | 4.9E-07 | 3.0E+14 | 7.8E+01 |
| CT6 | 3.8E+12 | 2.1E-02 | 3.0E-07 | 1.8E+14 | 4.7E+01 |
| CT7 | 3.1E+12 | 8.7E-02 | 1.2E-06 | 7.4E+14 | 2.4E+02 |
| CT8 | 2.7E+12 | 1.1E-01 | 1.6E-06 | 9.7E+14 | 3.6E+02 |
| CTD1.3 | 3.1E+12 | 8.1E-02 | 1.1E-06 | 6.8E+14 | 2.2E+02 |

^a^ determined by densitometry

^b^ labeled – unlabelled phage

^c^ Abs494/71000

^d^ [FITC] (M)*6.02x10^20^
